# Proteomic analysis of urine in medication-overuse headache patients: possible relation with renal damages

**DOI:** 10.1007/s10194-011-0390-9

**Published:** 2011-10-14

**Authors:** Elisa Bellei, Aurora Cuoghi, Emanuela Monari, Stefania Bergamini, Luca Isaia Fantoni, Maurizio Zappaterra, Simona Guerzoni, Annalisa Bazzocchi, Aldo Tomasi, Luigi Alberto Pini

**Affiliations:** 1 Medical Faculty, Department of Laboratory, Pathological Anatomy and Forensic Medicine, University Hospital of Modena and Reggio Emilia, Via del Pozzo 71, 41100 Modena, Italy; 2 Inter Department Headache and Drug Abuse Center, University Hospital of Modena and Reggio Emilia, Modena, Italy

**Keywords:** Medication-overuse headache, NSAIDs, Triptans, Kidney damage, Urinary proteomics

## Abstract

Medication-overuse headache (MOH) is a chronic disorder associated with overuse of analgesic drugs, triptans, non-steroidal anti-inflammatory drugs (NSAIDs) or other acute headache compounds. Various epidemiologic investigations proved that different drug types could cause nephrotoxicity, particularly in chronic patients. The aim of the present work was to analyze, by a proteomic approach, the urinary protein profiles of MOH patients focusing on daily use of NSAIDs, mixtures and triptans that could reasonably be related to potential renal damage. We selected 43 MOH patients overusing triptans (*n* = 18), NSAIDs (*n* = 11), and mixtures (*n* = 14), for 2–30 years with a mean daily analgesic intake of 1.5 ± 0.9 doses, and a control group composed of 16 healthy volunteers. Urine proteins were analyzed by mono-dimensional gel electrophoresis and identified by mass spectrometry analysis. Comparing the proteomic profiles of patients and controls, we found a significantly different protein expression, especially in the NSAIDs group, in which seven proteins resulted over-secreted from kidney (OR = 49, 95% CI 2.53–948.67 vs. controls; OR = 11.6, 95% CI 0.92–147.57 vs. triptans and mixtures groups). Six of these proteins (uromodulin, α-1-microglobulin, zinc-α-2-glycoprotein, cystatin C, Ig-kappa-chain, and inter-α-trypsin heavy chain H4) were strongly correlated with various forms of kidney disorders. Otherwise, in mixtures and in triptans abusers, only three proteins were potentially associated to pathological conditions (OR = 4.2, 95% CI 0.33–53.12, vs. controls). In conclusion, this preliminary proteomic study allowed us to define the urinary protein pattern of MOH patients that is related to the abused drug. According with the obtained results, we believe that the risk of nephrotoxicity should be considered particularly in MOH patients who abuse of NSAIDs.

## Introduction

The term medication-overuse headache (MOH) was recently introduced by the International Headache Society to describe daily or nearly daily (chronic) headache that occurs after the regular intake (overuse) of analgesic drugs, triptans, or other anti-headache or anti-migraine drugs [1, 2]; their excessive use for the treatment of pain conditions led to the development of MOH in about 1.7% of the people in Europe, Asia, and North America [3].

The molecular basis and the pathophysiology of MOH are still largely unknown, although it has been hypothesized that this condition could be mediated by cognitive impulsivity, and shares some dysfunction mechanisms with drug addiction [4], probably involving factors beyond the pain alone, such as certain behaviors and psychologic states [5]. Moreover, a combination of environmental and genetic factors may contribute to a patient’s vulnerability to intoxication, substance overuse, dependence and withdrawal in MOH [6, 7]. Recently, a role of dopamine-related genes in the genetic liability to chronic headache with drug abuse has been proposed, suggesting transmitted susceptibility to medication overuse [8, 9]. Therefore, an involvement of the serotonin transporter gene in the development of analgesics overuse in chronic tension-type headache patients has been postulated [10]. Hence, MOH has been investigated in both clinical and experimental studies, in the attempt to define its clinical picture, features and biologic basis. To date, however, no studies have been carried out to evaluate the possible relationship between MOH and potential renal injury, although drug-associated nephrotoxicity accounts for 18–27% of all acute kidney injury cases in the USA [11]. Currently, the most pertinent applications in nephrotoxicology can be found in the proteomic analysis of renal drug effects [12] and in the study of the consequences on the kidney induced by environmental toxins, drugs and other bioactive agents, analyzing urine [13]. Human urine is a useful biologic fluid for clinical proteomics study, as it can be collected easily and non-invasively in large quantities, and because it is a stable sample compared with other biofluids. It has been defined as a fluid biopsy of the kidney and urogenital tract, so many changes in these organs may be detected in urine [14, 15]. Urinary proteomics has thus become one of the most attractive subdisciplines in clinical proteomics, particularly for biomarker discovery [16] and clinical diagnostics [17].

The aim of this project was to analyze the urinary proteome of patients with MOH, in comparison with healthy subjects, with the purpose to identify possible differences in excreted proteins induced by excessive consumption of NSAIDs, mixtures and triptans that could be related to nephrotoxicity. We adopted a proteomic approach that is one-dimensional sodium dodecyl sulfate-polyacrylamide gel electrophoresis (1D-SDS-PAGE) in conjunction with mass spectrometry (MS) analysis, to discover and identify potential early urinary biomarkers able to predict probable kidney damages in MOH patients.

## Materials and methods

### Patients selection

Forty-three MOH patients were recruited by the “Headache and Drug Abuse Center” and the “Unit of Toxicology and Clinical Pharmacology” of the University Hospital of Modena and Reggio Emilia. As shown in Table 1, they were divided into three groups according to the type of primary abused drug: patients who consumed exclusively triptans (16 women and 2 men), aged 32–65 years (mean 45.7 ± 10.8), exclusively NSAIDs (11 women), aged 36–62 years (mean 49.8 ± 7.9), and patients (13 women and 1 man) assuming mixtures, in 90% of case containing indometacin, caffeine and perchlorperazine, aged 35–66 years (mean 56.7 ± 11.8).

Healthy volunteers (12 women and 4 men), aged 37–65 years (mean 46.2 ± 5.4), with a history of normal renal function were also enrolled and used as controls. For all study participants, exclusion criteria included: (1) proved kidney diseases and other acute or chronic medical illness, (2) elevated serum creatinine levels, and (3) prescription and consumption of counter medicines (other than NSAIDs and triptans for MOH patients). In fact, none of the patients and controls had hypertension, inflammatory diseases, acute or chronic medical illness, heart failure, malignancy, and renal dysfunctions. Routine laboratory analyses were carried out in all the subjects, including serum creatinine, uric acid, urinary pH and urine specific gravity. The study was approved by the Research Ethics Committee of the University Hospital of Modena, and informed consent was provided by all volunteers and patients.

### Urine samples collection and preparation

Taking into account that urine samples were investigated by proteomic methods, first of all we followed proper and standardized procedures for urine collection, preparation and storage [18].

All subjects were asked to refrain from unusual physical activity the day before urine collection, to avoid transient increase in protein excretion. Second void morning urine samples were collected, discarding first jet, but not the final (midstream). The urine was collected into a sterile polypropylene container, and immediately placed on ice. To remove cell debris and cellular contamination, urine samples were centrifuged at 800×*g* for 10 min at 4°C, then, the supernatant was divided into aliquots and stored at −80°C. In normal conditions, human urine has a very diluted protein concentration (usually does not exceed 10 mg/100 mL), with a high-salt content. For these reasons, before proteomic analysis, 4 mL of urine were concentrated and desalted using 3 kDa MW-cut off filter devices (Millipore). By this procedure urine was concentrated about 50-fold. Total protein concentration was estimated by the spectrophotometric Bradford’s method [19].

### One-dimensional gel electrophoresis (1D-SDS-PAGE)

Sodium dodecyl sulfate-polyacrylamide gel electrophoresis (SDS-PAGE) was performed according to Laemmli’s procedure [20], under reducing conditions. Urine samples were pooled and 10 μg of total proteins for each group were mixed with the Laemmli sample buffer (62.5 mM Tris–HCl, pH 6.8, 25% glycerol, 2% SDS, 0.01% bromophenol blue) plus 0.5% dithiotreitol (DTT). Sample mixtures were boiled at 95°C for 5 min, and then denaturated samples were loaded in duplicate onto 10 well 12% SDS-PAGE. To ensure optimal band resolution, the electrophoretic run was carried out in a minigel slab apparatus (Bio-Rad), using TGS running buffer (25 mM Tris–HCl, 192 mM glycine, 0.1% SDS, pH 8.3). Electrophoresis was initially run at 100 V for 30 min, followed by an increase up to 200 V, until the dye front reached the bottom of the gel. Finally, urinary proteins were visualized by incubation for at least 4 h in colloidal Coomassie Blue G-250, under gentle shaking, and later destained with 5% acetic acid. To verify the experiment reproducibility, distinct gels were stained with a more sensitive silver nitrate staining protocol, as previously described in detail [21].

### Image analysis

Gel images were acquired by a calibrated densitometer (Bio-Rad GS800) and analyzed by the powerful 1-D image analysis software program “Quantity One” (Bio-Rad). This software allows the identification of differentially expressed proteins in the different groups, and accurately detects increased or decreased proteins on the basis of spots staining intensity. The imaging device supported by this software is a light detector that converts signals from biologic samples into digital data, and subsequently displays the data in the form of a gray-scale. The total intensity of protein band is obtained by the sum of the intensities of all the pixels that make up the band, and the signal intensity is expressed as optical density (OD). Lane-based quantitation involves calculating the average intensity of pixels across the band’s width and integrating over the band’s height. In order to compensate for differences in intensity between lanes and correct the variability due to the staining methods, the band volumes were normalized as a percentage of the total OD of all the bands present in the gel. Moreover, to minimize background and noise density in the image maintaining data integrity, a lane-based background subtraction was performed. The protein bands were quantitatively, qualitatively, and statistically analyzed.

### Mass spectrometry analysis

The bands of interest were excised from the gels and analyzed by a quadrupole-time of flight liquid chromatography-mass spectrometry (Q-TOF LC/MS). Briefly, protein bands were de-stained using acetonitrile, reduced with DTT and alkylated with iodoacetamide, and subsequently digested with trypsin overnight at 37°C. After digestion, the peptides were first extracted with acetonitrile/ammonium bicarbonate, followed by a second extraction with formic acid. Finally, the pooled peptides extracted were concentrated in a vacuum drier and examined using the 6520 Accurate-Mass Q-TOF LC/MS (Agilent Technologies Inc., CA, USA), as previously fully described [22].

### Statistical analysis

Student’s *t*-test, with selected level of significance set at the probability value <0.05, and Bonferroni correction were used to compare demographic and clinical data of MOH patients and control group (Table 1). Odds ratio (OR) and risk ratio (RR) were used to evaluate non-parametric values of protein profiles (Table 3), because in control subjects and in triptans and mixtures abusers most bands were not detectable. In both tables, all data are provided as mean ± standard deviation (SD).

## Results

Demographic and clinical characteristics of MOH patients and control subjects are reported in Table 1. Data were compared using the Student’s *t*-test and the Bonferroni correction. The unique significant difference was related to age, and was found in the mixtures group vs. control and triptans groups.

**Table 1:** Descriptive demographic, headache and clinical data of control subjects and MOH patients

	Control subjects (*n* = 16)	Triptans group (*n* = 18)	NSAIDs group (*n* = 11)	Mixtures group (*n* = 14)	Total patients (*n* = 43)
Age (years)	46.2 ± 5.4*	45.7 ± 10.8*	49.8 ± 7.9	56.7 ± 11.8	48.4 ± 10.5
Gender (F/M)	12/4	16/2	11/0	13/1	40/3
BMI	26.0 ± 3.5	25.3 ± 4.4	23.4 ± 4.4	25.9 ± 6.4	25.1 ± 5.0
DDI	*NA*	1.2 ± 0.4	1.9 ± 1.4	1.6 ± 0.9	1.5 ± 0.9
MOH duration (years)	*NA*	7.5 ± 5.8	8.6 ± 7.9	9.0 ± 9.6	8.5 ± 7.6
LDQ	14.3 ± 4.2	15.7 ± 4.5	14.3 ± 6.1	17.2 ± 4.2	15.5 ± 4.8
SBP (mmHg)	116.4 ± 12.7	116.7 ± 15.2	110 ± 10.1	119.2 ± 16.3	115.7±14.1
DBP (mmHg)	70.2 ± 8.5	71.1 ± 11.7	70.0 ± 10.1	71.7 ± 11.7	71.0 ± 10.7
Serum creatinine (mg/dL)	0.6 ± 0.2	0.8 ± 0.1	0.7 ± 0.1	0.7 ± 0.2	0.7 ± 0.1
Serum uric acid (mg/dL)	4.0 ± 1.1	3.8 ± 0.9	3.6 ± 1.3	4.3 ± 1.6	3.9 ± 1.2
Urinary pH	5.3 ± 0.7	5.7 ± 0.8	5.5 ± 0.7	6.2 ± 1.1	5.7 ± 0.9
Urine specific gravity	1013 ± 5	1018 ± 6	1014 ± 6	1012 ± 5	1015 ± 6

Data are expressed as mean ± SD. Statistical significance was evaluated using Student’s *t*-test (* *P* < 0.01 vs. mixtures group. *P* = 0.008 after Bonferroni correction)

Groups: triptans (exclusively one, or more types of triptans), NSAIDs (exclusively one, or more types of NSAIDs), mixtures (consumption of drugs containing indometacin, caffeine and sedatives)

*BMI* body mass index, *DDI* daily drug intake, *MOH* medication-overuse headache, *LDQ* Leed’s drugs questionnaire, *SBP* systolic blood pressure, *DBP* dyastolic blood pressure

**Fig. 1:**
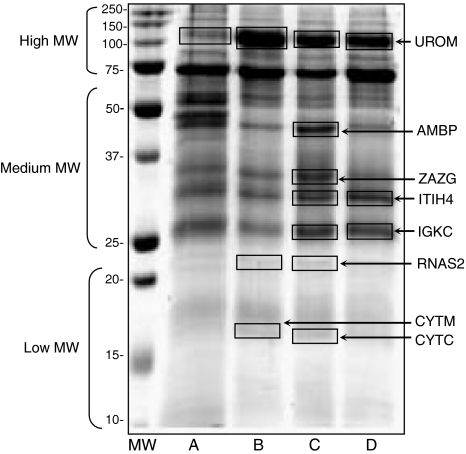
SDS-PAGE profiling of urinary proteins from control subjects (*lane A*), triptans (*lane B*), NSAIDs (*lane C*), and mixtures (*lane D*) abusers. *MW* molecular weight marker ladder (Precision Plus protein standard, Bio-Rad). In *boxes* are enclosed the differential protein bands among groups, and on the right side of the figure are reported the entry names of the proteins identified by MS analysis; *UROM* Uromodulin, *ITIH4* Inter-α-trypsin inhibitor heavy chain H4, *ZAZG* Zinc-α-2 glycoprotein, *AMBP* α-1-microglobulin, *IGKC* Immunoglobulin kappa chain C region, *RNAS2* Non-secretory ribonuclease 2, *CYTC* Cystatin-C, *CYTM* Cystatin-M

In order to characterize the urinary proteomic profile of each group, urine proteins were separated by SDS-PAGE analysis, according to their molecular weight (MW), as shown in Fig. 1. Excellent reproducibility among the various experiments was indicated by an accurate and total overlapping of the definite protein pattern obtained from duplicate gels. Moreover, Coomassie Blue staining gave the same results as silver nitrate staining protocol (data not shown).

**Fig. 2:**
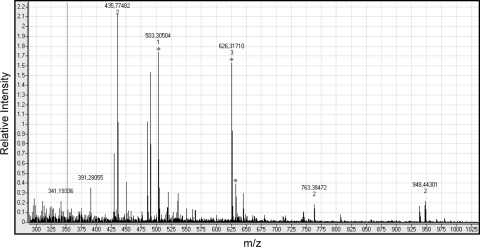
An example of a representative mass spectrum (obtained after ionization of UROM protein by Q-TOF LC/MS), illustrating the distribution of ions by mass-to-charge ratio (*m*/*z*) and relative abundance (intensity)

Comparing the patients proteomic profiles with those of healthy controls, we revealed a different protein expression at various MW levels (OR = ∞). The significantly different bands were excised from the gels and analyzed by Q-TOF LC/MS, after trypsin digestion. A representative mass spectrum concerning the identification of UROM is illustrated in Fig. 2. The protein sample was ionized producing charged molecules (ions), which were separated on the basis of their mass-to-charge ratio (*m*/*z*) in an electromagnetic field, and then the ion signals produced were processed into mass spectra. All the identified proteins are listed in Table 2. In column 1 are reported the protein entry names derived from the UniProt knowledge database, all with extension “Human”. Entry names corresponded to those indicated in Fig. 1. Column 2 denotes the UniProt database primary protein accession number, column 3 the recommended and commonly used protein name and column 4 the gene name. Column 5 refers to the probability based ions scores, associated with the queries. Ions score is −10 × Log(P), where P is the probability that the observed match between the experimental data and the database sequence is a random event. Individual ions scores >25 indicate identity or extensive homology. Protein scores are derived from ions scores as a non-probabilistic basis for ranking protein hits. The query values designate the number of peptides that match the identified protein (setting at least ten matching peptides). Column 6 shows the sequence coverage, namely the percentage of amino acids sequenced for each detected protein, and the last column displays their main function. The listed proteins were selected considering the highest scores, queries, and the greater amino acids sequence coverage among all the proposed identifications.

**Table 2:** Differentially expressed proteins identified by Q-TOF LC/MS analysis

Entry name	Accession n°.	Protein name	Gene name	Score/queries	Cov. (%)	Primary function
UROM	P07911	Uromodulin (or Tamm-Horsfall urinary glycoprotein)	UMOD	8131/525^a^	41^a^	Regulation
				2781/192^b^	32^b^	
				7532/480^c^	39^c^	
AMBP	P02760	Alpha-1-microglobulin	AMBP	1218/161^b^	56^b^	Inhibition
ZAZG	P25311	Zinc-alpha-2-glycoprotein	AZGP1	589/53^b^	30^b^	Lipid degradation
ITIH4	Q14624	Inter-α-trypsin heavy chain H4	ITIH4	500/63^b^	42^b^	Acute phase reaction
				390/71^c^	50^c^	
IGKC	P01834	Ig kappa chain C region	IGKC	2396/141^b^	93^b^	Immune response
				2609/142^c^	93^c^	
RNAS2	P10153	Non-secretory ribonuclease	RNASE2	571/51^a^	21^a^	Multifunction
				468/44^b^	21^b^	
CYTM	Q15828	Cystatin-M	CST6	80/10^a^	28^a^	Protease inhibitor
CYTC	P01034	Cystatin-C	CST3	172/13^b^	23^b^	Inhibition/Regulation

*Entry name* UniProt knowledge database entries, all with extension _HUMAN. Entry names corresponded to those reported in Fig. 2, *Accession n°* primary accession number from UniProt database, *Score:* the highest scores obtained using MASCOT search engine, *Queries* number of peptides that match the identified protein (at least ten matching peptides), *Cov.* (coverage) percentage of amino acids sequenced for each detected protein

^a^values detected in triptans group

^b^values detected in NSAIDs group

^c^values detected in mixtures group

By subjecting protein bands to densitometry with the Quantity One software (Bio-Rad), it turned out that the total OD in lane A (controls) was 24,000, whereas that of lane B (triptans) was 20,500, for lane C (NSAIDs) was 49,200, and for lane D (mixtures) was 35,500. Noteworthy is that the total OD of proteins present in NSAIDs lane was twice as much, in abundance, in comparison with controls. The mean OD of each differential protein band is reported in Table 3. The more significant differences, as also clearly evident in Fig. 1, emerged within the group of healthy controls (lane A) and NSAIDs patients (lane C), since seven proteins (enclosed in rectangle) resulted over-secreted from kidney: uromodulin (UROM), α-1-microglobulin (AMBP), zinc-α-2-glycoprotein ZAZG), inter-α-trypsin heavy chain H4 (ITIH4), Ig kappa chain C region (IGKC), non-secretory ribonuclease (RNAS2), and cystatin-C (CYTC) (OR = 49, 95% CI 2.53–948.67; RR = 7, 95% CI 1.09–44.60 vs. controls OR = 11.6, 95% CI 0.92–147.57; RR = 5, 95% CI 0.74–33.77 vs. triptans and mixtures groups). In particular, at high MW, all patients groups showed a very intensive protein band, corresponding to UROM, which was much less visible in controls. At medium MW (range 25–70 kDa), ITIH4 plus IGKC, and RNAS2 resulted up-regulated not only in the NSAIDs group, but also in mixtures abusers (lane D) and in triptans patients (lane B), respectively (OR = 4.2, 95% CI 0.33–53.12; RR = 1.4, 95% CI 0.77–2.54 vs. controls). Finally, at low MW (11–20 kDa), the CYTM was found up-regulated only in the triptans group.

**Table 3:** Optical densities of differentially expressed proteins in control subjects and MOH patients

	Control group	Triptans group	NSAIDs group	Mixtures group
UROM	2,205 ± 291	14,800 ± 2,764	12,500 ± 1,652	11,100 ± 982
AMBP	ND	ND	9,650 ± 1,393	ND
ZAZG	ND	ND	6,580 ± 969	ND
ITIH4	ND	ND	5,760 ± 941	7,560 ± 746
IGKC	ND	ND	6,200 ± 876	5,480 ± 1,216
RNAS2	ND	1,230 ± 231	1,450 ± 181	ND
CYTM	ND	1,125 ± 192	ND	ND
CYTC	ND	ND	1,340 ± 198	ND

Optical densities, detected by “Quantity One” 1-D image analysis software (Bio-Rad), were expressed as means ± standard deviation. Seven proteins were over-secreted in NSAIDs group: OR = 49, 95% CI 2.53–948.67; RR = 7, 95% CI 1.09–44.60 vs. controls; OR = 11.6, 95% CI 0.92–147.57; RR = 5, 95% CI 0.74–33.77 vs. triptans and mixtures groups. Three proteins were over-secreted in triptans and mixtures groups: OR = 4.2, 95% CI 0.33–53.12; RR = 1.4, 95% CI 0.77–2.54 vs. controls

*ND* protein not-detectable in the sample

In addition, four bands around 50 kDa were most evident in lane A (control group) compared to patient groups, and were identified as albumin fragments (data not shown). Furthermore, the intense band above (75 kDa), present in all groups, corresponded to serum albumin (ALBU, Acc. No. P02768). In urine, albumin exists in two forms: native (a single polypeptide) and a modified form comprised of large fragments. Infact, it is well demonstrated that filtered albumin is excreted as an heterogeneous population of albumin-derived molecules resulting from extensive degradation (>90%) during renal passage, to produce fragments that are excreted in urine [23].

## Discussion

At first, some data shown in Table 1 need to be discussed to clarify the studied population. Among the groups there was a difference in age only in the mixture users versus triptans and control subjects. This difference could be explained by two observations: firstly, patients assuming analgesic mixtures are historically older, when the clinical practice for treatment of chronic headache was based on the use of classic mixtures, as association of caffeine, phenothiazines or barbiturates and NSAIDs. The second one is that the use of mixtures comes after the incoming loss of efficacy of simple NSAIDs, so these are the patients with longer histories of headaches. All the other clinical and headache parameters did not differ significantly within groups. Interestingly, there were no differences between users of mixtures, drugs with a noticeable addiction risk, and triptans, which have never shown potential addictive activity in animal tests. Moreover, all blood and urine analysis were in the normal range, also in individuals with 30 and more years of daily drug intake.

Urine samples are used to study renal physiology and kidney diseases, as it contains useful biologic protein markers. Actually, disorders which adversely affect the function of the kidney can cause impaired proteins reabsorption and consequently their excessive losses in the urine. Direct nephrotoxic consequences may occur both in glomerular and tubular cells, as a result of different mechanisms, such as disruption of normal cellular functions, induction of intratubular obstruction, cellular swelling and tubular luminal occlusion [24].

One feasible approach to address renal drug effects is the employment of multiparametric tests, e.g., the innovative genomics, metabolomics and proteomics based assays that enable the simultaneous assessment of several parameters. During the past few years, proteomics has been extensively applied to various fields of medicine, including nephrology [25]. Particularly, proteomic analysis for nephrotoxicity was first introduced by Aicher et al. [26] to determine the association between renal protein changes and cyclosporine A nephrotoxicity in renal transplant patients. Afterwards, some proteomic studies were conducted on rat urine, e.g., to find out renal effects of puromycin aminonucleoside [27], or, more recently, of *cis*Platin, to discover drug-induced renal cytotoxicity biomarkers [28].

While adverse effects from long-term triptans use are unknown, the overuse of analgesics may cause well-known unwanted events, including liver dysfunction, gastrointestinal bleeding, renal insufficiency, and addiction [29]. Several research studies developed over the past 15 years reported the evidence of NSAIDs-induced kidney dysfunctions, such as acute renal failure and electrolyte imbalance. Nonetheless, the exact mechanism(s) of nephrotoxicity remains still unclear, though most theories focus on the initial inhibition of the cyclooxygenase (COX), the enzyme involved in prostaglandins (PG) synthesis, producing vasoconstriction and the subsequent perturbation of the numerous actions of COX in the kidney [30].

Despite all this evidence, at present no studies have yet been performed in the attempt to search for possible early biomarkers of kidney damages in MOH patients. Remarkably, these patients represent a specific model because they do not have any chronic systematic diseases, such as immunologic and cardiovascular disorders or hypertension; hence, this population is a model of healthy subjects who assume daily doses of analgesic drugs from many years. In this perspective, we can consider the use of NSAIDs and triptans as the unique possible cause of kidney impairment, and its early modifications can be investigated by proteomics.

In the present study we defined a preliminary and basic urinary proteomic profile of MOH. The most evident and unquestionable result is the finding of a marked presence of UROM in all patients groups, compared with control subjects (Fig. 1). UROM (also known as Tamm–Horsfall protein) is a large glycoprotein exclusively synthesized in the kidney and secreted into the urine via proteolytic cleavage. Its physiologic role is still not fully established. Nevertheless, it seems very likely that this protein is involved in the development of cast nephropathy, formation of renal stones, immunologic defence in the kidney, as well as in the modulation of systemic immunologic events [31]. Urinary UROM has also been postulated as a suitable parameter for determining the functional state of the kidney. Very recently, polymorphisms in the UROM gene have been found responsible for increased urinary UROM production and for an elevated risk to develop chronic renal alterations, such as the so-called familial juvenile hyperuricemic nephropathy, medullary cystic kidney disease and glomerulocystic kidney disease [32]. Other identified proteins which resulted over-secreted from kidney in MOH-NSAIDs abusers include AMBP, ZAZG and CYTC. AMBP is a stable urinary indicator protein, which reflects acute and chronic dysfunctions of the proximal renal tubule. This protein has an immunomodulatory role, with a broad spectrum of possible clinical applications, besides being a promising marker for evaluation of tubular function [33]. High concentrations in urine were found to be indicative of tubular drug toxicity, interstitial nephritis or chronic renal failure. Furthermore, determination of urinary AMBP has been used to screen for nephropathy due to environmental hazards, like intoxications with heavy metals [34]. Regarding ZAZG, Kumar et al. [35] detected this protein, by proteomic analysis, in urine of patients with different renal diseases, such as nephrotic syndrome, kidney failure and microalbuminuria. They supposed that ZAZG, together with other tubular damage markers identified in their study (including AMBP), could probably to be suggestive of first stages of renal tubular injury. Lastly, CYTC has been widely proposed as an accurate biomarker of glomerular filtration for the early detection of acute kidney injury (AKI) [36]. CYTC is a low-MW protein that, due to its relatively small size, normally is freely filtered at the glomerulus and then proteolytically digested and/or completely reabsorbed along the nephron. Tubular damage in AKI impairs this latter process and allows for this protein to appear in the urine, thus making it potentially suitable as a marker of AKI [37]. Recently, it was reported that immunohistochemical CYTC expression in the proximal tubule was altered by some glomerular and/or tubular nephrotoxicants in rats [38]. Moreover, we discovered other two proteins, namely ITIH4 and IGKC, clearly associated with NSAIDs and mixtures overuse. ITIH4 is involved in the pathogenesis of calcium oxalate lithiasis and has been recently linked to urinary stones disease [39], while immunoglobulin free light chains may cause kidney injury affecting all its compartments, in a broad variety of disease patterns [40]. Finally, regarding RNAS2 (found in both NSAIDs and triptans abusers), and CYTM (revealed only in triptans), so far no correlation have been reported between these proteins and renal disorders.

These findings suggest that the consumption of a mixture of drugs seems to be safer than the use of NSAIDs with regards to the possibility to induce nephrotoxicity in MOH patients. This is even more evident for triptans, since in this group we detected only one protein potentially related with renal toxicity. Actually, based on the available literature, all the 6 proteins that we found significantly higher in urine of NSAIDs abusers (excluding RNAS2) have demonstrated a reasonable correlation with different types of kidney dysfunctions. However, it is important to outline that all the study participants did not show any sign of renal damage, and in our clinical practice we did not register patients who developed renal impairment, except in a case with concomitant arterial hypertension.

In conclusion, proteomic technology proved to be a promising tool for the characterization of the urinary proteome in nephrotoxicologic research, and its application in this field may provide prognostic, therapeutic and monitoring guidance for MOH patients. This study offers the basis for subsequent perspective works directed toward better defining this problem and to confirm and extend the present results, using further specialized proteomic strategies.
